# Understanding Factors Associated with Interest in Sustainability-Certified Wine among American and Italian Consumers

**DOI:** 10.3390/foods13101468

**Published:** 2024-05-09

**Authors:** Mingze Rui, Federica Rosa, Andrea Viberti, Filippo Brun, Stefano Massaglia, Simone Blanc

**Affiliations:** 1 Department of Agricultural, Forest, and Food Sciences, University of Turin, 10095 Grugliasco, Italy; mingze.rui@unito.it (M.R.); filippo.brun@unito.it (F.B.); stefano.massaglia@unito.it (S.M.); 2 RV Studio s.r.l., 12051 Alba, Italy; federica@rvstudio.eu (F.R.); andrea@rvstudio.eu (A.V.); 3 Centro Interdipartimentale Viticoltura e Vino (CONViVi), University of Turin, 12051 Alba, Italy

**Keywords:** sustainability, wine, consumer study, Italian wine market, US wine market

## Abstract

The wine industry has been witnessing a growth in businesses crafting sustainability-certified wines and in the attention of consumers to sustainability, especially in the United States and Italy. To identify the characteristics of consumers who prefer sustainability-certified wine, this study analysed the relationship between consumers’ demographics, wine buying behaviour, and interest in sustainability-certified wine, focusing on these two countries for comparison. Data were collected through an online survey of US and Italian consumers. Through correspondence analysis, *k*-modes clustering analysis, and multi-way correspondence analysis, this study revealed a stronger relationship between demographics and interest in sustainability-certified wine among US consumers than Italian consumers. In particular, middle-aged US consumers exhibited a greater interest than seniors. The patterns of connections between consumers’ wine buying behaviour and interest in sustainable wine were similar for the two countries. In particular, consumers who purchase wine weekly had a keen interest, and those who purchase wine sporadically had no or little interest. Furthermore, this study uncovered the intricate relationship among various variables, providing a comprehensive understanding of the association between wine consumer characteristics and their interest in sustainability-certified wine.

## 1. Introduction

In recent years, the global wine industry has witnessed a significant growth in wine businesses with a sustainability initiative for its linkage to long-term profitability [1]. The industry has been facing threats from climate change and water and energy scarcity, leading to the adoption of sustainable production plans and socially responsible behaviour [2]. The increasing environmental concerns have called attention to the environmental dimension of sustainability in the wine industry, and both consumers and businesses are highly interested in eco-friendly and organic wines and processes [3]. As the leader in both wine consumption and importation in the world [4], the US market has undoubtedly become the focus of wine merchants, particularly due to the increasing attention of US consumers to sustainability in their wine choices [5]. Meanwhile, Italy, ranking third in wine consumption and first in wine production [4], has opted to actively promote sustainability in recent years [6], especially under the evolving political landscape of the European Union [7]. Italian wine consumers have also shown a rising interest in sustainably produced wine [8]. The comparison between the US and Italy, representing new world and traditional world wine countries, respectively, has been a subject of numerous research studies exploring consumer behaviour [9], market trends [10,11], and business strategies [12]. Recent studies have delved into topics such as premium pricing for organic wine [13], Italian consumers’ acceptance of fungus-resistant grape wines [14], and the impacts of sustainable packaging on wine sales [15]. Other studies have examined consumers’ perception of the designation of origin [9] and the characteristics of US consumers interested in wines from vineyards using cover crops [16]. Nonetheless, a comprehensive understanding of wine consumers’ choices of wines with sustainability attributes remains to be fully uncovered [17], especially within these two prominent wine markets.

### 1.1. Sustainable Wine Certifications in the US and Italy

Sustainability, as defined by United Nations (UN), entails ‘meeting the needs of the present without compromising the ability of future generations to meet their own needs’ [18]. In the wine industry, sustainability involves a wide range of practices; according to the International Organisation of Vine and Wine (OIV), sustainable vitiviniculture aims to preserve natural resources and improve socio-economic conditions through balanced consideration of environmental, social, and economic aspects with appropriate planning, assessment, and communication [19]. Three important types of sustainable wine are organic, biodynamic, and natural wine; they each encompass distinct principles and practices. Among these, organic wine production is well defined and regulated by legislation in some countries such as the United States, Canada, India, Japan, and countries of the European Union [20]. Biodynamic wine production, closely aligned with the sustainability approach, builds upon organic practices and shares many of their principles [21]. However, the definition and standards of biodynamic wine production still lack public intervention. Some certification schemes exist at international level, and the most popular biodynamic certifications with well-established standards are provided by organisations like Demeter International and Union Biodyvin. Natural wine also lacks a strict definition; however, efforts to establish certifications for natural wines are actively underway in some countries, such as France’s recent recognition by the Institut National de l’Origine et de la Qualité (INAO) in 2020 [22].

Since the establishment of the first sustainable winegrowing program by the Californian Lodi Winegrape Commission in 1992 [20], numerous other programs worldwide have emerged, each setting its own standards. Unlike organic, biodynamic, and natural wine focusing on environmental sustainability, sustainable vitiviniculture offers a holistic guideline tailored to local ecosystems and socio-economic conditions. In the US, the Certified California Sustainable Vineyard and Winery (CCSW), the LODI RULES for Sustainable Winegrowing Certification Program, and SIP (Sustainability in Practice) Certified are prominent wine sustainability certification schemes. Some other important regional certification schemes are LIVE (Low Input Viticulture and Enology) Certified and the Salmon-Safe certification. In Italy, the program ‘Sustainability in the Italian wine sector (VIVA)’ is the only Italian public standard for measuring the sustainability performance of the vine–wine chain and has been proved to outperform the European benchmarks regarding the environmental sustainability of the wine sector [23,24]. Moreover, the National Integrated Production Quality System (SQNPI) is gaining popularity among wine growers and producers. Additionally, international programs like Fairtrade International and Fair for Life focus on fair pricing and improved social conditions for producers and communities.

### 1.2. Consumer Behaviour in Sustainable Wine Consumption

Consumer studies in sustainable wine reveal multifaceted insights into consumers’ perceptions, preferences, and behaviours regarding environmentally friendly wine products. These studies explore various factors influencing consumer choices, including eco-certifications [25,26,27], demographic characteristics [27,28,29,30,31], and product attributes [32,33,34,35]. Research indicates that consumers are increasingly integrating environmental considerations into their wine purchasing decisions [36], with willingness to pay premiums for eco-certified wines emerging as a notable trend across different markets [25,26,27]. Clustering analysis identifies distinct consumer segments based on beliefs about environmental protection, attitudes towards sustainability, geographical locations, and WTP for sustainable-labelled wines, highlighting the diverse range of preferences within wine consumer populations [25,36]. Generational differences also play a significant role in driving consumers’ attitudes and purchasing intentions to sustainable wine [27,28]. Additionally, factors such as organic production methods [30,32,34], sulfite content [33,35], and attitudes towards healthy eating and the environment influence consumers’ WTP for specific wine attributes [33,34,35]. These findings underscore the importance of understanding consumer attitudes and preferences to effectively market sustainable wine products and promote environmental stewardship within the wine industry.

Consumers’ attitudes towards sustainable wine are shaped by a multitude of factors, as highlighted in recent systematic reviews of wine consumer studies [17]. Moderator variables such as age, income, gender, generation, culture, personality traits, and country play significant roles in influencing wine consumption [17], particularly in the context of sustainability. Sustainable wine consumption is not only about the taste or quality of the wine but also about the ethical and environmental considerations associated with its production and distribution. Therefore, understanding how moderator variables intersect with sustainability attitudes and behaviours is crucial for both researchers and practitioners in the wine industry.

Consumers’ interest in sustainable wine is evident from consistent findings across research studies. Numerous studies have revealed consumers’ positive perceptions of sustainable wine and a willingness to pay a price premium [37]. The willingness of consumers to pay a premium for sustainable wines implies potential economic benefits for wine businesses [14]. Research has revealed demographic influences on consumers’ choices of sustainable wine. In particular, younger generations showed a more positive attitude towards wines with sustainability attributes compared to their older counterparts [34,38,39]. Furthermore, millennial consumers tended to pay a high premium price for these wines [28,40]. Specifically, among this demographic, millennial males have been identified as possessing strong environmental attitudes towards wine products [31]. Varied attitudes among young Italian wine consumers towards sustainably produced wine have also been observed [41]. Moreover, gender was another important factor influencing consumers’ attitudes to sustainable wine, specifically in shaping consumers’ willingness to pay a premium for sustainable wine [36]. Research has identified that female consumers are more positive about sustainable wine than male consumers [27,30], but there was also a study which found that gender was not a factor influencing preferences for sustainable wine [33]. As environmental consciousness continues to rise, consumers are increasingly integrating environmental considerations into their wine purchasing decisions [36]. Notably, early research on environmentally friendly wine revealed that consumers’ awareness of environmental issues correlated more strongly with their engagement in broader environmental concerns than with their self-assessed wine knowledge [42]. Interestingly, consumers with strong normative beliefs demonstrated a willingness to pay a premium for pro-environmental wines, driven by the belief that their purchases contribute positively to the environment [43]. Furthermore, recent research has indicated that consumers with high involvement in sustainable wines are typically better educated and more involved in the wine culture [39].

Additionally, researchers have identified a link between higher wine purchasing frequency and a greater willingness to pay for sustainable wine [29], although some studies suggest occasional wine buyers may demonstrate a higher willingness to pay than regular wine buyers [27].

Despite these insights, most studies have focused on consumers from single countries, overlooking potential cultural variations in preferences [17]. There is also a lack of comprehensive research analysing consumer characteristics, including demographics, geographical locations, and wine buying behaviours, among individuals interested in sustainably certified wines [2]. These gaps highlight the need for further research to gain a deeper understanding of the characteristics of wine consumers inclined towards the sustainability attributes of wine.

### 1.3. Aims and Scope of This Study

This study aims to address several gaps in existing research. Firstly, while prior studies often focus on consumers from single countries, neglecting potential cultural variations in preferences, this study conducts comparative analyses between the US and Italian wine markets to elucidate cross-cultural differences in consumer attitudes towards sustainability-certified wine. Secondly, previous research has primarily examined consumer attitudes towards sustainable wine without comprehensively analysing various factors regarding demographic characteristics, geographical locations, and wine buying behaviours. This study seeks to fill this gap by exploring the complex interplay among these factors and consumers’ inclination towards sustainability-certified wine. Specifically, this study achieves these objectives by addressing two fundamental research questions (RQs):

- RQ1. How do the demographic characteristics and buying behaviour of wine consumers in the US and Italy associate with their interest in sustainability-certified wine?
- RQ2. To what extent do interactions exist among the demographic and wine buying behavioural characteristics of wine consumers, their geographical locations (i.e., the US and Italy), and their inclination towards sustainability-certified wine?

RQ1 aims to identify the association of an individual variable (i.e., demographic characteristics or buying behaviour) with consumers’ inclination to sustainability-certified wine. RQ2 focuses on understanding the complex relationships among multiple variables influencing consumers’ interest in sustainability-certified wine. Through these inquiries, this study seeks to provide valuable insights into the factors driving consumer interest in sustainable wine within the US and Italian wine markets.

## 2. Materials and Methods

### 2.1. Data Collection

In 2023, we collected data through an online survey of consumers’ interest in sustainability-certified wine among 1000 and 1250 individuals from the United States and Italy, respectively. The questionnaire was anonymous and did not include any sensitive information. Prior to participation, the respondents were asked to provide consent after reading a project disclosure sheet that outlined the objectives of the survey. The research adhered to the principles of the Declaration of Helsinki, and the only requirement for participating in the survey was that the participants had to be at least 18 years old. The survey contained questions about demographic information, wine consumption habits, and levels of interest in sustainability-certified wine. Table 1 provides a detailed breakdown of survey questions and responses. The dataset comprises three variables for each country: consumers’ demographic characteristics, wine buying behaviour, and their interest level in sustainability-certified wine, with each variable including five categories (gender, age, region, living arrangement, and occupation), four categories (wine buying frequency, habitual buying of imported or low-alcoholic wine, and the preferred consumption channel for EU wine), and one category (interest level), respectively. Each category features 2 to 4 levels of answers. To facilitate the use of statistical tools, each response was coded as shown in the last column in Table 1. Respondents were excluded from the analysis if they were younger than the legal age of alcohol consumption (21 years old in the US and 18 years old in Italy) in their respective country or if they were not a wine drinker (i.e., those who chose ‘never’ for wine purchase frequency). The final dataset contains a total of 2063 valid responses, with 864 participants from the US and 1199 from Italy.

### 2.2. Data Analysis

**Figure 1:**
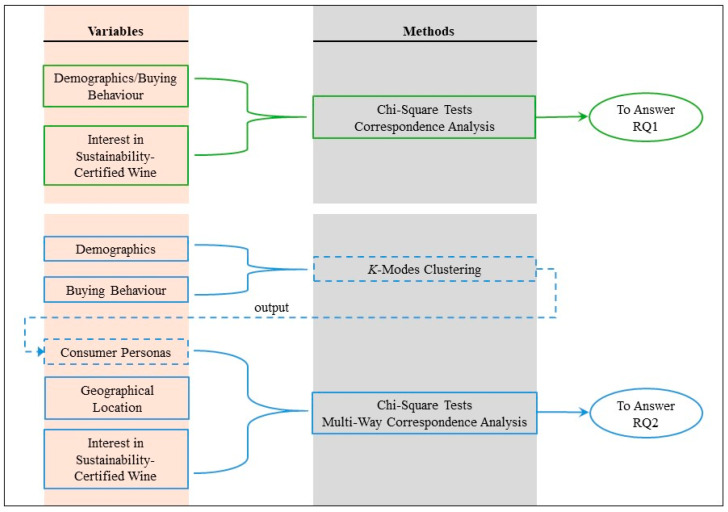
Methodological framework for answering research questions.

Figure 1 depicts the methodological framework designed to answer the two RQs. Symmetric Correspondence Analysis (CA) was conducted to answer RQ1. CA has been widely used in exploring relationships among complex categorical variables. It transforms two-way and multi-way tables into visually interpretable 2-D maps, facilitating the analysis of intricate categorical datasets [44,45,46]. In this study, firstly, chi-square tests examined the independence of demographic characteristics and sustainability interest in the US and Italy, using a significance level (α) of 0.05. Secondly, generalised singular value decomposition (GSVD) was applied to the Pearson residuals. Two-dimensional correspondence plots, using principal coordinates, were generated. To discern the individual contribution of each variable category to the statistical significance of the overall association, *p*-values were generated for each point (i.e., each category) in each analysis, using a significance level (α) of 0.05. The same analyses were repeated for buying behaviour and sustainability interest in each country.

A mixed-methodology approach (Figure 1) was designed to answer RQ2: given the abundance of variable categories, *k*-modes clustering was used to generate consumers’ personas based on their demographic characteristics and buying behaviour; following this, multi-way correspondence analysis (MWCA) was applied to consumers’ personas, geographical location (i.e., the US or Italy), and their interest in sustainability-certified wine.

*K*-modes clustering has emerged as a specialised technique designed explicitly for the effective handling of categorical data [47,48]. Unlike *k*-means, which utilises the mean as a centroid measure, *k*-modes employs a mode-based approach to clustering [49]. The selection of initial cluster modes is crucial for the algorithm’s performance, and various improvements, such as novel dissimilarity measures and strategies for handling boundary data, have been proposed to enhance its efficiency and accuracy [48,49,50,51,52]. Experimental studies have demonstrated the superiority of these enhanced algorithms in effectively clustering categorical datasets [53,54]. *K*-modes clustering has increasingly been applied in recent research across diverse fields, playing a crucial role in analysing categorical data for tasks such as market segmentation, customer profiling, and pattern recognition [49,55,56,57,58,59].

MWCA is a variant of CA; it is a visualisation method applied to a multi-way contingency table by reserving its cube or hypercube structure. A common variant of CA, multiple correspondence analysis (MCA), is widely used to visualise data in a multi-way contingency table using singular value decomposition (SVD). This method involves the transformation of data with a cube or hypercube format to a two-way matrix, which sometimes oversimplifies data and causes unacceptable loss of information [60]. To solve this problem, some multi-way versions of SVD were developed, one of which was designed to effectively handle data with a three-way format is the Tucker3 decomposition model (T3D) [61]. In this study, MWCA was employed to explore the relationship among wine consumers’ personas, geographical location, and their inclination to sustainability-certified wine.

Specifically, *k*-modes clustering grouped individuals (including all respondents of the two countries) with similar profiles consisting of all categories of consumers’ demographics and buying behaviour with the exception of their residing regions (e.g., Northern Italy) within their respective countries. The determination of optimal cluster numbers was achieved using the Elbow method based on the total dissimilarities (i.e., cost) of clusters through a representative *k*-number plot. For MWCA, a T3D was applied to the three-way Pearson residuals by using the maximum number of components. The first two dimensions for each variable were extracted for visualisation. To discern potential interactions, chi-square tests of independence were conducted for all pairwise variables (e.g., demographic characteristics and buying behaviour) and the trio of variables. Among these, the paired variables that contributed most significantly to the overall association were identified and then used to generate biplots for visualisation. For a clear illustration of methodology, we call such paired variables an ‘interactive duo’ and the other variable an ‘unpaired variable’ in this study. Such biplots, called interactive biplots, were defined by Beh and Lombardo in Section 7.4.2 in their book [46], with two types of biplots particularly proposed for the assessment of associations among three variables. In our case, both two biplots were produced, and their descriptions are as follows:

- Biplot 1: interactive biplot employing the paired variables (i.e., the ‘interactive duo’) as the projection with the other variable (i.e., the ‘unpaired variable’) plotted by the principal coordinates.
- Biplot 2: interactive biplot employing the principal coordinates of the interactive duo with the unpaired variable serving as the projection.

Beta (β) scaling, defined by the Equations (1) and (2) below, proposed by Beh and Lombardo [46], may be applied to coordinates for a clearer visualisation:

(1) $${\tilde{f}}_{is}=\beta f_{is}$$

(2) $${\tilde{g}}_{jks}=\frac{g_{iks}}{\beta },$$

where $f_{is}$ stands for principal coordinates of the *i*th category of the unpaired variable in the sth dimension; $g_{iks}$ stands for the combined principal coordinates of the *j*th category of one of the interactive duo and the kth category of the other variable of the interactive duo in the sth dimension; *β* is the scaled value, and it is positive; ${\tilde{f}}_{is}$ and ${\tilde{g}}_{jks}$ are the scaled principal coordinates of the unpaired variable and the interactive duo, respectively. The inner products of the biplot coordinates were also summarised to interpret the results of biplots more precisely. R Studio (Version 2023.09.2+508) [62] was used to assist the data analysis.

## 3. Results

This study identified the connections between consumers’ interest in sustainability-certified wine and their characteristics in terms of demographics and wine buying behaviour through multiple methods of categorical data analytics and compared such connections between US and Italian consumers. The remaining part of the Results section is guided by different types of methods, with Section 3.1 addressing RQ1 and Section 3.2 and Section 3.3 addressing RQ2.

### 3.1. Symmetric Correspondence Analysis

#### 3.1.1. Results of Chi-Square Tests of Independence

Table 2 shows the results of the chi-square tests of independence of consumer demographics and wine buying behaviour in relation to the level of interest in sustainability-certified wine in the US and Italy. The results show that both demographic characteristics and buying behaviour have associations with sustainability interest for both countries. It is worth mentioning that among the four tests, the results for demographics in Italy indicated the weakest association compared to others.

#### 3.1.2. The Two-Dimensional Correspondence Plots

**Figure 2:**
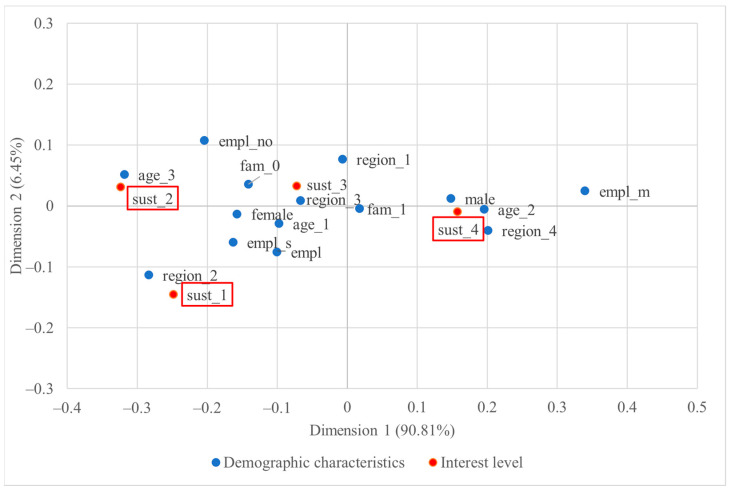
Two-dimensional symmetric correspondence plot of US consumers’ demographic characteristics and level of interest in sustainability-certified wine; statistically significant points are highlighted with rectangles.

The two-dimensional correspondence plot of US consumers’ demographic characteristics and level of interest in sustainability-certified wine is depicted in Figure 2, visually summarising 97.26% of total inertia. The visualisation provides a high-quality representation of the associations, as over 70% can be considered as good quality [46].

In the US, visual analysis of the plot (Figure 2) indicates that consumers from the Midwest region (region_2) tended to exhibit the least interest in sustainability-certified wine (sust_1), but those from the West had an intense interest (sust_4). Similarly, the senior demographic group (age_3) did not show much interest in sustainability-certified wine (sust_2), but the middle-aged one expressed a keen interest (sust_4). Moreover, male consumers may be more interested in such wine than the female consumers. However, it is important to note that none of the demographic categories made a statistically significant contribution to the observed association, at least within a 95% confidence interval. Even though some of these categories (e.g., age_3, empl_m) were located far from the origin, the insignificant results could be due partially to a small number of respondents in these categories. On the other hand, three specific interest levels (sust_1, sust_2, and sust_4) significantly contributed to the total association.

**Figure 3:**
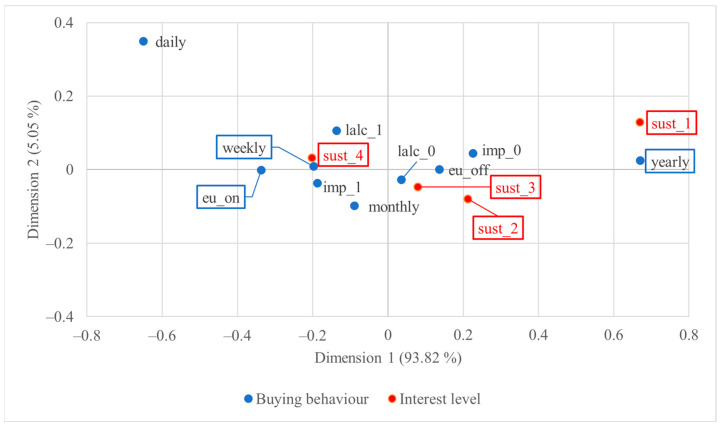
Two-dimensional symmetric correspondence plot of US consumers’ wine buying behaviour and level of interest in sustainability-certified wine; statistically significant points are highlighted with rectangles.

Figure 3 below shows the correspondence plot of US consumers’ wine buying behaviour and their interest in sustainability-certified wine. The visualisation represents 98.87% of the total inertia, with dimension 1 alone representing 93.82% of the total inertia, which signified the great contribution of association by the horizontal distance between points.

Based on the visualisation of the CA plot in Figure 3, a robust association was revealed between annual or occasional wine purchase behaviour and minimal sustainability interest. Conversely, weekly wine drinkers and individuals who habitually purchase imported wine exhibited intense interest in sustainability-certified wine. Moreover, individuals who predominantly consume EU-origin wine outside their homes and those who habitually purchase imported wine exhibited greater interest compared to their respective counterparts. The statistically significant points further proved these trends.

**Figure 4:**
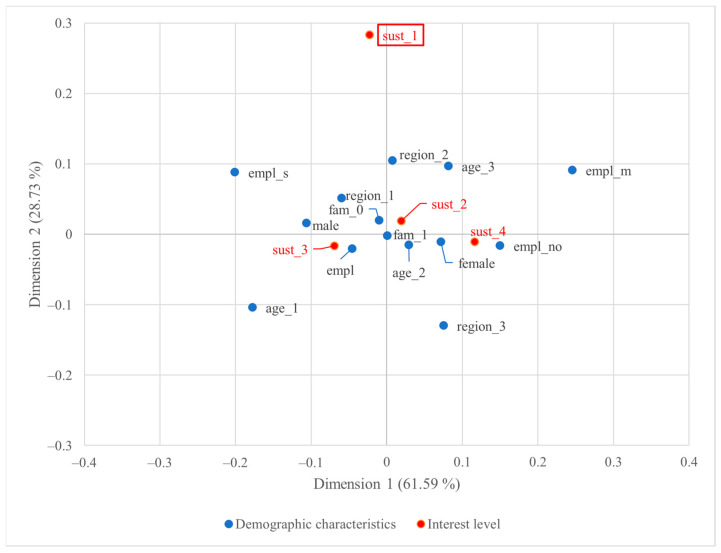
Two-dimensional symmetric correspondence plot of Italian consumers’ demographic characteristics and level of interest in sustainability-certified wine; statistically significant points are highlighted with rectangles.

The two-dimensional correspondence plot of Italian consumers’ demographic characteristics and their sustainability interest is presented in Figure 4. The visualisation summarised 90.31% of the total association.

For Italian consumers, the majority of plots (Figure 4) are closely clustered around the origin, indicating a weak association between demographic characteristics and their interest in sustainability-certified wine. This is consistent with the result of the chi-square test, as discussed in Section 3.1.1. Only one variable category, negligible or minimal interest in sustainability-certified wines (sust_1), reached statistical significance, further confirming the inference of the weak association. Even so, the distance between points in the plot indicates that individuals without employment may have greater interest compared to their counterparts, and those from Central Italy may have no or little interest but not those from other regions.

**Figure 5:**
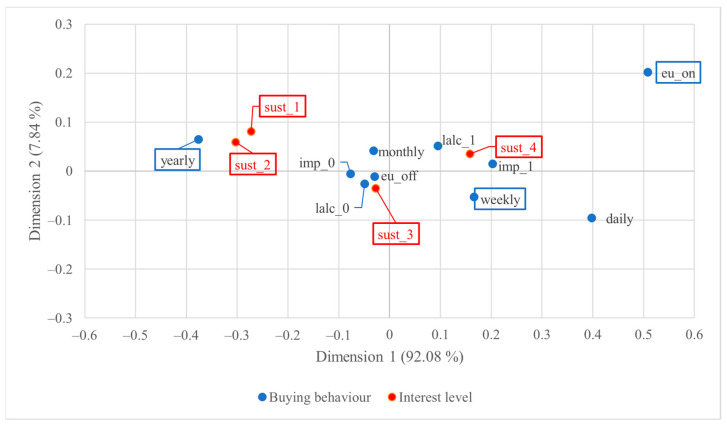
Two-dimensional symmetric correspondence plot of Italian consumers’ wine buying behaviour and level of interest in sustainability-certified wine; statistically significant points are highlighted with rectangles.

Figure 5 presents the correspondence plot of Italian consumers’ wine buying behaviour and their interest in sustainability-certified wine. The visualisation represents 99.93% of the total association, which is nearly 100%, indicating an almost complete presentation of total association; most of the association is presented by the horizontal distance between points.

In the analysis of Italian consumers’ buying behaviour and their sustainability interest, some results mirrored those observed in the analysis of US consumers. Those who consume wine yearly or occasionally exhibited less interest in sustainability-certified wine compared to those who purchase wine weekly. Moreover, individuals who habitually buy imported or low-alcoholic wine demonstrated a pronounced interest in sustainability-certified wine. In particular, all four levels of sustainability interest are statistically significant. Similar to the results for the US, the three variable categories yearly or occasional wine purchases, weekly wine purchases, and habitual EU wine consumption through on-trade channels significantly contributed to the observed statistical associations between buying behaviour and sustainability interest. These consistent patterns underscore the robustness of these factors in influencing the link between buying behaviour and consumers’ interest in sustainability-certified wine for both Italian and US consumers.

### 3.2. K-Modes Clustering

Four clusters were formed, and the demographic characteristics of consumers and their wine buying behaviours for each cluster are summarised in Table 3. The most representative response of each category for each cluster is highlighted (Table 3). Based on the representative responses for each cluster, the characteristics of each cluster in terms of consumers’ demographics and buying behaviours can be concluded, forming a distinctive persona for each cluster.

The four personas are described as follows: The first persona is labelled ‘Occasional Wine Connoisseur’. This persona typifies a middle-aged male who is employed in a non-managerial position and lives with his family. He partakes in social drinking sporadically and favours imported wines. The second persona, ‘Executive Wine Enthusiast’, portrays a middle-aged male in a managerial role, living with family, and engaging in weekly wine consumption. This persona exhibits a preference for imported wines and often explores EU wine through on-trade channels. The third persona, ‘Wise Wellness Explorer’, characterises a senior male who is occupying a non-managerial role and lives with family. He enjoys occasional wine sipping, particularly favouring low-alcoholic options. The fourth persona, ‘Vibrant Female Drinker’, portrays a young female employed in a non-managerial role. She purchases wine weekly with a preference for domestic wines.

### 3.3. Multi-Way Correspondence Analysis

Table 4 shows the distribution of consumers by their persona types, residing country, and their interest in sustainability-certified wines. The majority of consumers fell into persona type 4, while persona type 2 had the fewest respondents overall. In Italy, persona 4 constituted the majority of respondents, while persona 2 had the smallest representation. Most Italian consumers showed a moderate to high level of interest in the sustainability of wine, with a minority expressing minimal interest. In particular, persona 4 with a moderate to high level of interest predominated among Italian consumers. For the US consumers, persona 2 had the highest number of respondents, followed by persona 4 with only a one-person difference. Interestingly, persona 2 rarely expressed minimal interest in sustainability irrespective of the respondents’ country of residence.

The partition of chi-squared of Table 4 is shown in Table 5. All chi-square test results for pairwise variables and the trio were significant, among which the pairwise variables, geographical location and persona types, contributed most to the total association with about 54% of total inertia.

**Figure 6:**
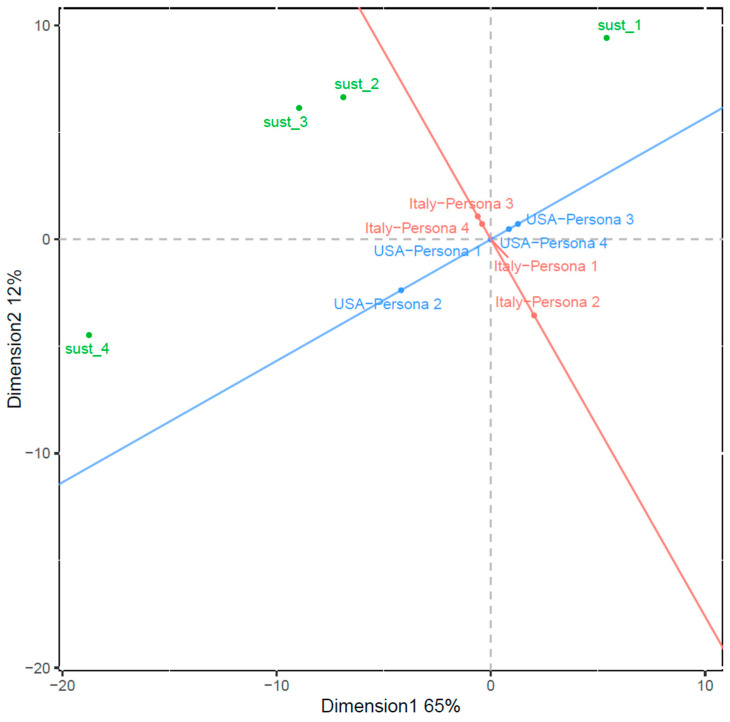
Biplot 1: interactive biplot of first two dimensions from the symmetric multi-way correspondence analysis with beta scaling (β = 0.5) using geographical location and persona type as the projection; consumer interest in sustainability-certified wine is displayed using principal coordinates.

**Figure 7:**
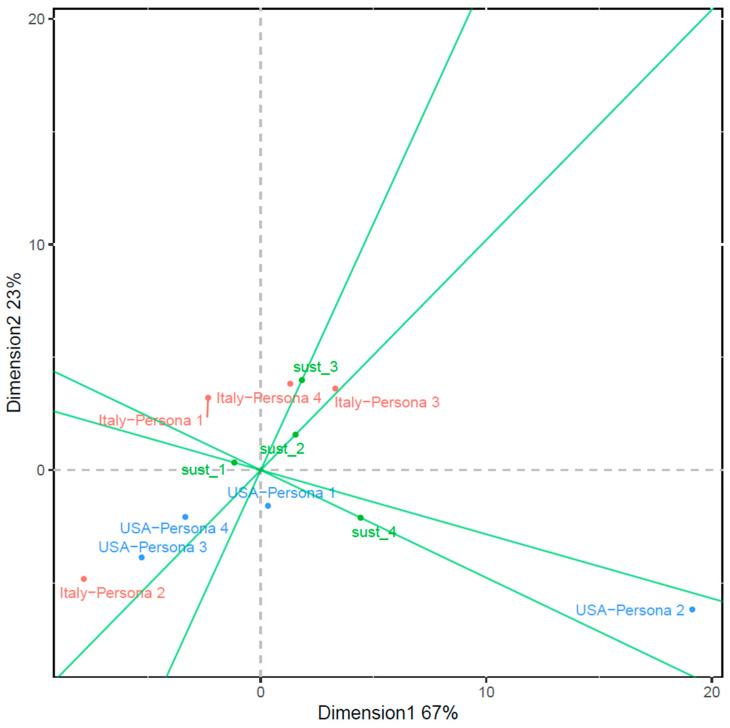
Biplot 2: interactive biplot of first two dimensions from the symmetric multi-way correspondence analysis with beta scaling (β = 3) using consumer interest in sustainability-certified wine as the projection; geographical location and persona type are displayed as the paired interactive variables using principal coordinates.

Therefore, two interactive biplots were produced using geographical location and persona type as the paired interactive variables (i.e., the ‘interactive duo’) and interest level in sustainability-certified wine as the unpaired variable. Biplot 1 and 2 are displayed in Figure 6 and Figure 7, respectively. Biplot 1 captures 77% of the total inertia, while biplot 2 represents 90% of the total inertia, making both good-quality visualisations of association, with biplot 2 demonstrating higher quality.

In biplot 1 (Figure 6), the four sustainability-certified wine interest levels stand prominently away from the origin, implying their significant contributions to the association of the three variables. Notably, except for the proximity between ‘sust_2’ and ‘sust_3’, the distances between these levels are considerable. Specifically, ‘sust_1’ and ‘sust_4’ exhibit the most substantial separation, highlighting significant differences between consumers with extremely low and high levels of interest in sustainability-certified wine regarding their persona types and geographical locations.

Of particular interest is ‘sust_4’, which is the closest point among the four interest levels to the projection of ‘USA-Persona 2’, suggesting the strongest connection of US Executive Wine Enthusiasts with intense interest in sustainable wine among all consumer types. Moreover, associations were observed for US Wise Wellness Explorers and Vibrant Female Drinkers with minimal interest. For Italy, Italian Wise Wellness Explorers and Vibrant Female Drinkers tended to have a low to moderate level of interest.

In biplot 2 (Figure 7), all paired variables, excluding ‘USA-Persona 1’, exhibit considerable distance from the origin, with ‘USA-Persona 2’ particularly standing out. This indicates that the interactions between these persona types and geographical locations had substantial impacts on the overall association among the three variables. Mirroring the pattern observed in biplot 1, biplot 2 confirms that the US Executive Wine Enthusiast maintained a strong connection with a very high level of interest in sustainable wines, while the Italian Wise Wellness Explorer was more associated with a low to moderate level of interest. Additionally, the Italian Vibrant Female Drinker showed the strongest association with a moderate to high level of interest.

In quadrant 3 (Figure 7), the points ‘Italy-Persona 2’, ‘USA-Persona 3’, and ‘USA-Persona 4’, despite their distance from the origin, are notably far from the projections of all four interest levels. This indicates that the associations between these geographical locations and persona types were not strongly linked to specific interest levels.

While the two-dimensional biplot effectively illustrates the relationship among the three variables, it lacks the representation of information in the third and higher dimensions. Moreover, interpreting distances from points to projections can be subjective. To address this, the inner products were calculated from the coordinates of all three variables across all dimensions and are presented in Table 6 below. Large positive values in these inner products indicate a robust association among the three variables, vice versa, the larger the absolute value of the negative inner product, the weaker the association. The inner products that exceed the third quartile (10.79) of all 32 values are highlighted in the table.

The inner product results align closely with the interpretations drawn from the biplots, affirming that the US Vibrant Female Drinker, Italian Wise Wellness Explorer, Italian Vibrant Female Drinker, and US Executive Wine Enthusiast exhibited strong associations with low, low to moderate, moderate to low, and high levels of interest in sustainability-certified wine, respectively. Despite potential ambiguity in the proximity of certain points to biplot projections, the inner product analysis reveals strong associations. Specifically, this includes instances such as the US Occasional Wine Connoisseur with a low interest level, US Executive Wine Enthusiast with a low to moderate interest level, and Italian Wise Wellness Explorer with a moderate to high interest level.

## 4. Discussion

This study has explored the connections among consumers’ demographic characteristics, wine buying behaviours, and their interest in sustainability-certified wine in Italy and the US. Addressing RQ1, demographics played a significant role in shaping consumers’ wine sustainability interest in the US, with middle-aged consumers exhibiting keen interest and seniors showing less interest. This aligns with the findings of existing studies, for example, US millennial consumers had a higher willingness to pay than older respondents for eco-certified wines [27] and wines with sustainability attributes in the Californian wine market [28]. Some previous studies suggested that female consumers might be more interested in wines with sustainable characteristics in Italy [29] and the US [27]. However, based on the results in this study, no significant variation in interest was identified between female and male consumers in Italy, and the results for the US indicated that male consumers might exhibit a greater interest in sustainability-certified wine than female consumers. Such a conflict suggests that further investigations into the association of gender with consumers’ interest in wine with sustainability attributes are needed. The CA plots in this study also suggested potential associations between consumers’ interest in sustainability-certified wine and their occupations or country regions (e.g., Western US and Midwestern US). While the association between interest and residing region was not statistically significant in this study, previous research has suggested that residence may indeed influence the strength of respondents’ environmental attitudes [31]. Future research is recommended to explore and validate this association. The results for both Italy and the US highlighted a clear and consistent relationship between consumers’ buying behavioural characteristics and their interest in sustainable wines. In particular, consumers engaging in weekly consumption were highly interested in sustainability-certified wine, and those with yearly or occasional consumption showed little or no interest. Similar results were found in existing studies, for example, US consumers with weekly consumption were more positively impacted by wine labels with sustainability indicators than those with lower purchasing frequency [35], and the Italian wine consumers with weekly wine consumption had a higher willingness to pay than those with lower frequency for wines with sustainability attributes [25,29] or, in particular, natural wine [33].

For RQ2, our study showcased the intricate relationships among consumers’ personas, geographical locations, and their interest in sustainability-certified wine. Our study has filled a significant gap since no existing research so far has specifically investigated such relationships. By recalling the results of the two biplots and the inner products of variables, we can conclude that the US Executive Wine Enthusiasts were greatly interested in sustainability-certified wine, the US Occasional Wine Connoisseurs showed no or little interest, and the Italian Wise Wellness Explorers had somewhat great interest. It is worth mentioning that previous research has identified a correlation between US millennial males and a strong preference for environmentally friendly wines [31], a segment closely resembling the US Executive Wine Enthusiasts identified in our study. This underscores the consistency of consumer segmentation across studies and highlights the significance of targeting specific consumer groups for sustainable wine initiatives.

For wine sellers who target the Italian and US wine markets, it is recommended to consider customers’ wine buying frequency when promoting sustainability-certified wine. Specifically, focusing on customers with a weekly wine purchasing habit may yield more effective results compared to those with a yearly or occasional purchasing habit. The country-specific features identified in this study hinted that wine marketers should tailor their approaches to different markets. In particular, promoting sustainable wine to consumers who habitually purchase low-alcoholic wines may be effective in Italy but not in the US. Moreover, wine sellers should consider comprehensive consumer profiles, integrating various demographic and buying behaviour characteristics, to formulate effective marketing strategies for wines with sustainability characteristics. For instance, promoting such wine to those who have a similar profile to US Executive Wine Enthusiasts rather than other consumer archetypes identified in this study may prove more successful. Therefore, maintaining updated customer information, including demographics and purchasing behaviour, is essential for wine producers to tailor marketing strategies effectively for sustainability-certified wine.

Although the relationships among variables were identified, the reasons behind these connections were not investigated in this study. This demands future research to clarify these reasons and provide guidance for wine sellers and marketers to not only effectively promote sustainable wine to interested consumers but also encourage those initially uninterested. Moreover, treating interest level and wine purchase frequency as nominal categories instead of ordinal further hindered the profound understanding of their relationships. Despite these limitations, the significant results offer an opportunity for future research to address these challenges and delve deeper into the topics mentioned above.

## 5. Conclusions

This study applied CA, *k*-mode clustering analysis, and MWCA to investigate the factors associated with consumers’ interest in sustainability-certified wine in the US and Italy. This study’s comprehensive analysis revealed significant associations between consumers’ interest in sustainability-certified wine and their demographic profiles and wine purchasing behaviours in both the US and Italy. While demographic characteristics and buying behaviours varied in their strength of association, they consistently influenced consumers’ sustainability preferences. Geographical location emerged as a key factor, with distinct regional differences observed, particularly in the US. Despite these variations, this study identified four distinct consumer personas, shedding light on segments with varying levels of interest in sustainable wine. In particular, certain patterns, such as the preference for sustainability among weekly wine buyers and those purchasing imported wines, transcended cultural boundaries. This study addresses a significant research gap by exploring the relationship between various consumer characteristics and their interest in sustainability-certified wine. By identifying distinct consumer personas and uncovering consistent patterns across geographical locations, this study offers valuable insights for targeted marketing strategies. Ultimately, the findings have the potential to drive positive change within the wine industry by promoting sustainability initiatives and meeting the growing demand for sustainable wines among consumers in Italy and the US. Moving forward, future research could delve deeper into additional factors shaping sustainability preferences and conduct longitudinal studies to track evolving consumer trends in the dynamic landscape of sustainable wine consumption. Furthermore, future research could also expand its scope to include more countries, enabling a broader understanding of global trends in sustainability preferences within the wine industry.

## Figures and Tables

**Table 1:** Survey overview with response codes: demographic characteristics, buying behaviour, and sustainability interest for wine products among US and Italian consumers.

Variable	Category	Response	Response Code
Demographic characteristics	Gender	Female	gender_1
		Male	gender_2
	Age	18–35 (young adult)	age_1
		36–50 (middle-aged adult)	age_2
		50+ (senior adult)	age_3
	Residing region	Northeastern US; Northern Italy	region_1
		Midwestern US; Central Italy	region_2
		Southern US; Southern Italy	region_3
		Western US	region_4
	Living arrangement	Live alone	fam_0
		Live with other family member(s)	fam_1
	Occupation	Employed in a non-managerial role	empl
		Employed in a managerial role	empl_m
		Self-employed	empl_s
		Not currently employed (e.g., student, retired, unemployed, etc.)	empl_no
Buying behaviour	Wine purchase frequency	Daily wine purchases	daily
		Weekly wine purchases	weekly
		Monthly wine purchases	monthly
		Yearly or occasional wine purchases	yearly
	Imported wine consumption	Non-habitual consumption	imp_0
		Habitual consumption	imp_1
	Low-alcoholic wine consumption	Non-habitual consumption	lalc_0
		Habitual consumption	lalc_1
	European Union-origin wine consumption channel	Habitual consumption through on-trade channels (e.g., bars, hotels, restaurants, etc.)	eu_on
		Habitual consumption through off-trade channels (e.g., supermarkets, online platforms, wine shops, etc.)	eu_off
Level of interest in sustainability-certified wines	Level of interest in sustainability-certified wines	Negligible or minimal	sust_1
		Low to moderate	sust_2
		Moderate to high	sust_3
		Extremely high	sust_4

**Table 2:** Test of independence of US and Italian consumer demographics and wine buying behaviour in relation to level of interest in sustainability-certified wines.

Chi-Square Test Parameters	US		Italy	
	Demographics	Buying Behaviour	Demographics	Buying Behaviour
Chi-square (observed value)	134.883	212.776	68.727	99.635
Chi-square (critical value)	58.124	40.113	54.572	40.113
df	42	27	39	27
*p*-value	<0.0001	<0.0001	0.0023	<0.0001
Alpha	0.05	0.05	0.05	0.05

**Table 3:** Consumers’ demographic and wine buying behavioural characteristics in four clusters, where the percentage values of the most representative response within each cluster are highlighted.

Category	Response	Cluster 1	Cluster 2	Cluster 3	Cluster 4
Gender	Female	35.0%	28.9%	41.5%	82.9%
	Male	65.0%	71.1%	58.5%	17.1%
Age	18–35	9.1%	17.2%	8.1%	45.0%
	36–50	75.0%	69.3%	31.3%	34.4%
	50+	15.9%	13.5%	60.6%	20.6%
Living Arrangement	Live alone	13.0%	7.4%	10.0%	8.4%
	Live with other family member(s)	87.0%	92.6%	90.0%	91.6%
Occupation	Employed in a non-managerial role	60.2%	18.9%	53.4%	56.1%
	Employed in a managerial role	9.1%	57.6%	7.7%	7.5%
	Self-employed	13.6%	7.7%	10.7%	9.7%
	Not currently employed	17.0%	15.8%	28.3%	26.7%
Wine purchase frequency	Daily wine purchases	3.2%	3.4%	1.2%	1.8%
	Weekly wine purchases	29.1%	76.8%	22.3%	58.8%
	Monthly wine purchases	25.0%	17.8%	67.1%	18.0%
	Yearly or occasional wine purchases	42.7%	2.0%	9.5%	21.4%
Imported wine consumption	Non-habitual consumption	42.3%	21.5%	79.4%	78.1%
	Habitual consumption	57.7%	78.5%	20.6%	21.9%
Low-alcoholic wine consumption	Non-habitual consumption	83.0%	76.2%	33.9%	82.9%
	Habitual consumption	17.0%	23.8%	66.1%	17.1%
Main Channel for Consuming EU Wine	On-trade channels	4.3%	66.5%	4.4%	5.3%
	Off-trade channels	95.7%	33.5%	95.6%	94.7%

**Table 4:** Consumer distribution by personas, geographical location, and sustainable wine interest.

Geographical Location	Interest in Sustainability-Certified Wine	Persona Type				Grand Total
		Persona 1: Occasional Wine Connoisseur	Persona 2: Executive Wine Enthusiast	Persona 3: Wise Wellness Explorer	Persona 4: Vibrant Female Drinker	
Italy	sust_1	12	3	16	15	46
	sust_2	19	3	28	51	101
	sust_3	145	28	171	320	664
	sust_4	62	35	113	178	388
	Total	238	69	328	564	1199
USA	sust_1	25	0	8	28	61
	sust_2	21	17	11	36	85
	sust_3	74	85	38	109	306
	sust_4	82	178	46	106	412
	Total	202	280	103	279	864
Grand Total		440	349	431	843	2063

**Table 5:** Partition of chi-squared of consumer distribution by personas, geographical location, and sustainable wine interest.

Chi-Square Test Parameters	Term-IJ *	Term-IK *	Term-JK *	Term-IJK *	Term-Total
Chi-squared	84.147	297.778	101.566	65.91	549.4
Phi-squared	0.041	0.144	0.049	0.032	0.266
% of inertia	15.316	54.2	18.487	11.997	100
df	3	3	9	9	24
*p*-value	<0.001	<0.001	<0.001	<0.001	<0.001
X^2^/df	28.049	99.259	11.285	7.323	22.892

* I = geographical location; J = interest in sustainability-certified wine; K = persona type.

**Table 6:** Inner products from the symmetric multi-way correspondence analysis.

Geographical Location	Persona Type	Sustainability Interest			
		sust_1	sust_2	sust_3	sust_4
USA	Persona 1	73.39	11.99	−6.63	6.7
	Persona 2	−45.42	13.17	10.76	97.22
	Persona 3	−6.61	−14.72	−25.08	−15.57
	Persona 4	24.03	5.95	−15.6	−10.25
Italy	Persona 1	−4.33	−7.99	9.35	−17.02
	Persona 2	−32.47	−37.97	−32.09	−25.21
	Persona 3	10.52	10.89	20.52	7.42
	Persona 4	−18.61	7.02	17.67	−2.87

## Data Availability

The original contributions presented in the study are included in the article, further inquiries can be directed to the corresponding author.
